# Effects of 12-week exercise on Meteorin-like levels, inflammation, and functional capacity in older adults: Korean national aging project randomized controlled study

**DOI:** 10.1007/s41999-025-01272-2

**Published:** 2025-07-23

**Authors:** Parivash Jamrasi, Jun Hyun Bae, Wook Song

**Affiliations:** 1 Department of Physical Education, Seoul National University, Seoul, 08826 Republic of Korea; 2 Institute of Sport Science, Seoul National University, Seoul, 08826 Republic of Korea; 3 Institute on Aging, Seoul National University, Seoul, 03087 Republic of Korea; 4 School of Kinesiology, University of Michigan, Michigan, 48109 United States of America

**Keywords:** Aging, Exercise, Meteorin-like protein (Metrnl), Inflammation, Functional Capacity

## Abstract

**Purpose:**

Meteorin-like protein (Metrnl) is involved in regulating inflammation, metabolism, and muscle regeneration, making it a promising therapeutic target. Building on prior research, we investigated how exercise-induced changes in Metrnl relate to inflammatory markers, physical function, and cognitive performance. This randomized controlled trial examined the effects of a 12-week exercise intervention on circulating Metrnl, a novel biomarker, in 90 community-dwelling older adults (≥ 65 years).

**Methods:**

Participants were randomized into a walking group (WG), a combined resistance and walking group (RWG), or an active control group (CG). Intervention groups engaged in moderate-intensity exercise; walking based on age-specific step goals and resistance training twice weekly. Blood samples and assessments of physical and cognitive function were collected pre- and post-intervention.

**Results:**

79 participants successfully completed the study. After 12 weeks, serum Metrnl levels significantly increased in both RWG and WG compared to CG (WG vs. CG: *p* = .002; RWG vs. CG: *p* = .004). Metrnl changes were correlated with improvements in inflammatory markers (IL-6, *p* = .048; TNF-α, *p* = .040), physical activity (*p* = .041), and physical function (Timed Up & Go, *p* = .004; Five Times Sit to Stand Test, *p* = .008). Stronger associations were observed in the RWG, including cognitive gains (Stroop test, *p* = .040), enhanced handgrip strength (*p* = .036), and reduced fat mass (p*p*= .021). Timed Up & Go and Five Times Sit to Stand Test were the strongest predictors of Metrnl changes.

**Conclusion:**

These findings highlight Metrnl’s potential as a biomarker linking exercise to reduced inflammation and improved physical and cognitive outcomes in older adults, supporting its relevance in developing targeted exercise-based therapies.

**Supplementary Information:**

The online version contains supplementary material available at 10.1007/s41999-025-01272-2.

## Key summary points

**Aim:**

This study investigated whether a 12-week exercise intervention alters circulating Metrnl levels in older adults and how these changes associate with inflammatory markers, physical function, and cognitive performance.

**Findings:**

A 12-week exercise intervention significantly increased Metrnl levels in older adults, with these increases correlating positively with improvements in inflammatory markers, physical function, and cognitive performance, particularly in the combined resistance and walking group.

**Message:**

Exercise can increase Metrnl, a biomarker linked to reduced inflammation and improved physical and cognitive function in older adults, suggesting potential for Metrnl-targeted therapies to enhance training responses.

**Supplementary Information:**

The online version contains supplementary material available at 10.1007/s41999-025-01272-2.

## Introduction

Aging is characterized by a weakened immune system driven by chronic systemic inflammation, often accompanied by declines in skeletal muscle and cognitive function [1]. These changes contribute to reduced functional capacity, increased disease risk, and a higher likelihood of injuries [1]. Regular exercise, however, can counteract these effects by stimulating the release of cytokines from various organs (e.g., skeletal muscle, adipose tissue, neurons), which are essential for energy metabolism and act as potent anti-inflammatory agents, thereby promoting overall health and well-being. Additionally, exercise-induced physiological adaptations enhance muscle metabolism, strength, and function. They also improve memory and stimulate neurovascular adaptations, helping to counteract the adverse effects of aging [2, 3]. In contrast, a sedentary lifestyle impairs muscle regeneration, promotes intermuscular fat accumulation, and reduces satellite cell numbers, leading to muscle atrophy and sarcopenia [2]. Physical inactivity is also strongly associated with cognitive impairment, increasing the risk of neurodegenerative diseases such as dementia and Alzheimer’s disease [4]. One of the underlying mechanisms is chronic systemic inflammation, which contributes to both cognitive and physical deterioration. Conversely, increased physical activity (PA) is consistently linked to lower levels of pro-inflammatory biomarkers [5, 6].

Among these, C-reactive protein (CRP), interleukin-6 (IL-6), and tumor necrosis factor-alpha (TNF-α) are well-recognized markers of systemic inflammation and have been associated with reduced muscle and brain function, and metabolic diseases in older adults [7, 8]. This highlights the significant role of chronic inflammation in contributing to functional decline [9–13]. While the benefits of exercise are well established, understanding the molecular mechanisms involved is essential. Exerkines, including myokines and adipokines released during PA, mediate many of the systemic effects of exercise, such as metabolic regulation, inflammation control, and tissue repair. Elucidating these pathways may support the development of targeted strategies for healthy aging.

Among the emerging exerkines, Metrnl has garnered significant attention as a novel adipokine and myokine involved in the regulation of inflammation, metabolism, and muscle regeneration. Metrnl is a recently identified secreted protein, initially characterized as being predominantly produced by myeloid cells, but it is also expressed in endothelial cells, adipocytes, monocytes, myocytes, and various regions of the nervous system [14, 15]. Although research on Metrnl remains limited, accumulating evidence suggests its protective roles in modulating inflammatory immune responses [16–18], facilitating cardiac and skeletal muscle repair [19–21], promoting neurite outgrowth [22], and contributing to systemic health, particularly through its effects on metabolic and cardiovascular function [15, 18, 20–25]. Recent evidence suggests that Metrnl deficiency may contribute to heightened inflammatory responses, as lower serum Metrnl levels have been significantly associated with elevated TNF-α and IL-6 in individuals with inflammatory conditions, highlighting its potential role in regulating inflammation and metabolic function [16, 17, 20]. Notably, Baht and colleagues suggests that Metrnl modulates inflammatory pathways and exerts anti-inflammatory effects via STAT3 signaling in macrophages, and its deficiency impairs muscle regeneration [20]. Building on these findings, recent studies have suggested that aging may be associated with reduced Metrnl expression, with decreased levels observed in aged mice [20, 26]; Metrnl modulates age-related muscle pathways, enhancing repair and function [26], though further research is needed to confirm these effects. Extending these findings to human studies, a recent study by Wang and colleagues was the first to report that reduced serum levels of Metrnl are significantly associated with an increased risk of sarcopenia in older adults [27]. Moreover, following identification of Metrnl as a neurotrophic factor [23, 28], recent study demonstrated its presence in the cerebrospinal fluid of patients, confirming that Metrnl crosses the blood–brain barrier and contributes to vital functions in the brain and spinal cord [22]. Collectively, these findings underscore the critical roles of Metrnl in inflammatory immune regulation, muscle regeneration, and neural health, while also highlighting its potential as a clinically relevant biomarker for age-related muscle decline.

Emerging evidence indicates that Metrnl levels in adults are positively influenced by regular physical activity, yet their association with functional capacity remains unclear [29]; and investigations into Metrnl’s exercise response in both humans and animals are still limited. Supporting this, recent evidence reported that aged mice exhibited reduced Metrnl expression alongside declines in grip strength and running capacity [26]; while similar findings in older adults showed positive correlations between serum Metrnl levels and ASMI, grip strength, and gait speed [27]. Complementing these observations, research in human adults indicates that various forms of acute exercise, including resistance training [30], high-intensity interval training (HIIT) [31], and concurrent exercise (combining resistance and aerobic training) [32], significantly elevate both intramuscular Metrnl mRNA expression and circulating levels in young, healthy individuals. In overweight individuals, both a 6-week aerobic program in adolescents [33] and a 16-week combined resistance and aerobic training intervention in adults with diabetes [34] increased Metrnl levels, highlighting the role of exercise in modulating this biomarker in different age groups and health conditions. As Metrnl secretion varies by exercise intensity and contraction type (aerobic, resistance, concentric, eccentric), further research is needed to determine the optimal exercise modality, especially in older populations where data is lacking.

In our previous study [35], we evaluated the effects of walking alone or combined with resistance training on functional performance in older adults, including its sustained impact after a 12-week follow-up. According to the literature, walking is a widely preferred activity that enhances fitness [36, 37], while resistance training effectively mitigates age-related declines in neuromuscular function and capacity [38]. Given that both exercise modalities are cost-effective and easy to integrate into daily routines [36], they were strategically designed to address functional decline in older adults. Notably, the combined exercise program significantly improved physical and cognitive function, with lasting benefits over time [35]. Expanding on our previous study, the present study further investigates the physiological adaptations to the same exercise interventions within this cohort. Given the critical importance of maintaining functional capacity and mitigating age-related inflammation in older adults, and considering Metrnl's established roles in these processes, investigating its response to exercise in this vulnerable population is particularly pertinent. Despite growing interest in Metrnl, its precise role in human exercise physiology remains unclear. Although preliminary findings suggest its involvement in inflammation regulation, metabolic homeostasis, and tissue repair, there is a significant gap in the literature concerning the specific effects of structured exercise interventions on circulating Metrnl levels in community-dwelling older adults. Moreover, little is known about how these changes relate to clinically relevant outcomes, such as physical function, inflammatory markers, and cognitive performance. To address this gap, the present study is the first to evaluate changes in serum Metrnl levels following a 12-week exercise intervention in older adults. In addition, we examined the associations between Metrnl and key inflammatory markers (CRP, IL-6, and TNF-α), as well as its relationship with physical and cognitive function. With no approved pharmacological treatments for age-related functional decline or chronic low-grade inflammation, exercise remains a promising, non-invasive strategy. Increased physical activity is associated with reduced inflammatory biomarkers and enhanced healthspan [39]. This study aims to contribute to the understanding of Metrnl as a potential biomarker and mechanistic mediator of exercise-induced benefits in aging populations.

## Methods

### Participants

**Fig. 1:**
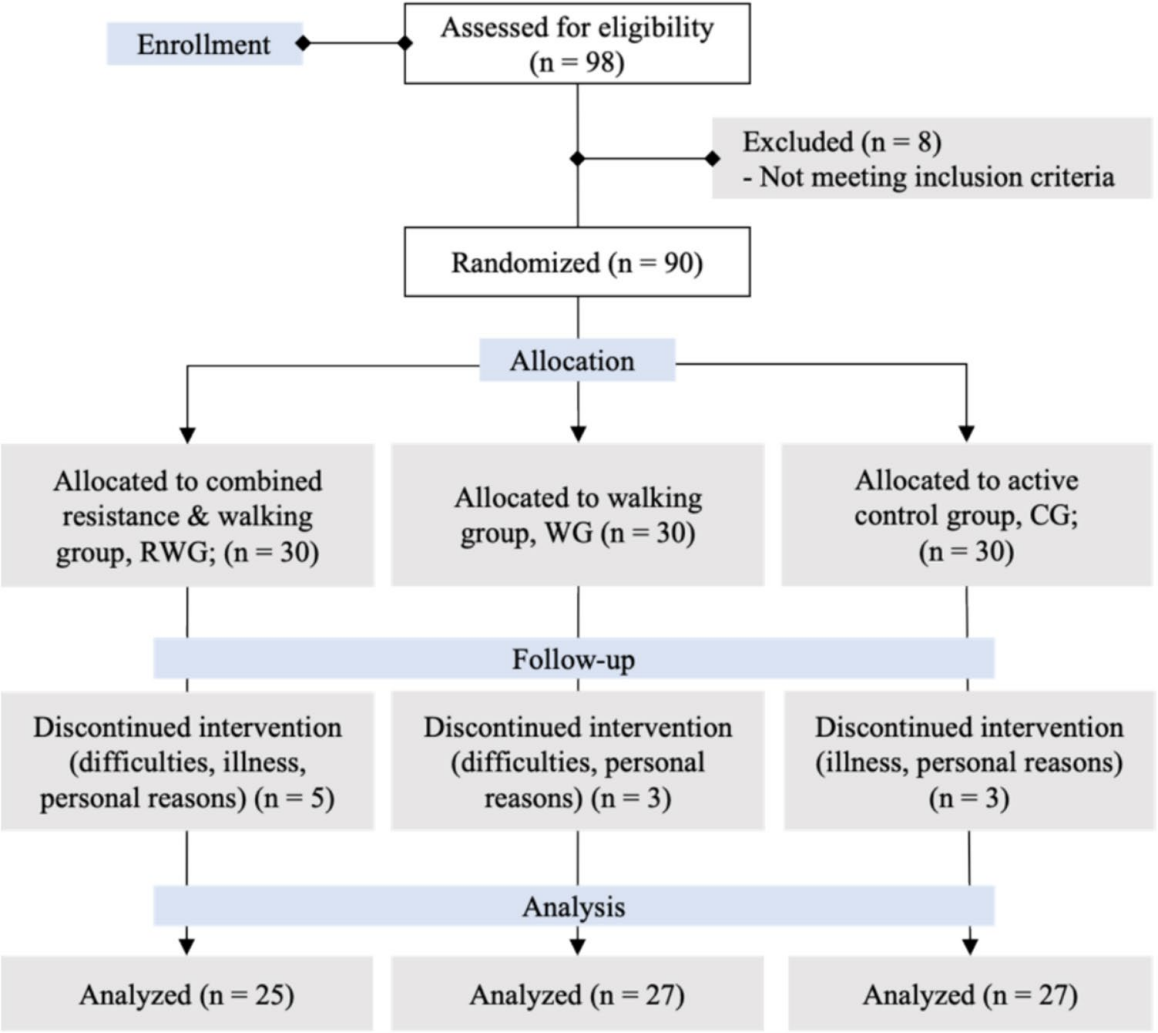
CONSORT flow diagram illustrating participant progression through the study

Ethical approval for this randomized controlled trial was obtained from the Institutional Review Board (SNUIRB No. 2106/002-009) and followed the protocol outlined in our previous study [35]. A total of 90 participants were recruited from senior centers near Seoul National University, based on a power calculation assuming 90% power, a 5% significance level, and a 20% anticipated dropout rate. Participants were excluded if they had engaged in high-intensity exercise prior to the study, had high aerobic fitness (indicated by a high VO₂peak), a body mass index (BMI) < 18.5 or > 30 kg/m^2^, or had a history of heavy alcohol or tobacco use, recent surgery, or supplement use. Participants were not involved in the design, conduct, or reporting of the study. After obtaining informed consent, 90 older adults aged ≥ 65 years were randomly assigned to one of three intervention groups: (1) combined resistance and walking group (RWG), (2) walking group (WG), and (3) active control group (CG) for a 12-week supervised intervention. Post-intervention measurements were conducted within three days of completing the intervention. The CONSORT 2025 flow diagram is outlined in Fig. 1.

### Intervention

The interventions were delivered by exercise physiologist with prior experience in conducting exercise interventions in older adult populations. The walking exercise protocol was designed as an individualized outdoor walking program, targeting a weekly step count with a minimum of two sessions per week, in line with national guideline and adjusted to moderate intensity according to NIH recommendations [40, 41]. The resistance protocol consisted of a 50-min, center-based progressive resistance training session using Thera-Band® twice weekly at moderate intensity [35]. The WG participated solely in the walking protocol, while the RWG engaged in both the walking and resistance protocols. Participants in both the WG and RWG were regularly monitored through phone check-ins and in-person visits throughout the study. In contrast, the control group maintained their usual exercise habits and was advised to perform only light-intensity stretching at home. Refer to the original publication for a detailed description of the study design and exercise protocol [35].

### Blood sampling and biochemical assessment

To identify target biomarkers, morning serum and plasma samples were collected by a professional phlebotomist before and after the intervention. All participants were informed about their 8-h fasting requirements. We centrifuged the samples at 3000 rpm for 10 min at 4 °C and stored them at − 80 °C until analysis. We measured serum Metrnl concentrations using a commercial ELISA kit (DuoSet ELISA, R&D Systems, catalog no. DY7867-05, Minneapolis, MN, USA) with intra- and inter-assay coefficients of variation (CVs) of < 8% and < 12%, respectively. Inflammatory cytokines were analyzed using ELISA kits from Invitrogen (Thermo Fisher Scientific, Waltham, MA, USA): Human TNF-α (catalog no. 88-7346-88) in plasma (intra-assay CV < 6%, inter-assay CV < 10%), Human IL-6 (catalog no. 88-7066-88) in serum (intra-assay CV < 5%, inter-assay CV < 9%), and Human C-Reactive Protein (catalog no. KHA0031) in plasma (intra-assay CV < 4%, inter-assay CV < 6%). We performed all ELISA assays in duplicate, according to the manufacturer’s instructions. The absorbance was measured at 450 nm using Magellan Software V7.2 (Tecan, Switzerland) with a Tecan Infinite Series M200 Pro microplate reader (Tecan, Switzerland), and we calculated the concentrations based on standard curves fitted with a four-parameter logistic (4PL).

### Functional capacity measurement tools

We assessed PA volume and metabolic equivalents (MET) using the Global Physical Activity Questionnaire (GPAQ) [42]. To measure body composition (muscle and fat mass), we used bioelectrical impedance analysis (InBody 720, InBody Co. Ltd, Seoul, Korea). To evaluate cognitive function, we administered the Stroop test, which includes color, word, and color-word conditions. We measured functional capacity using the 5 times sit-to-stand (5 × SST), timed up and go (TUG), and electronic short physical performance battery (eSPPB, Dyphi Inc., Daejeon, Korea). Additionally, we assessed handgrip strength (HGS) with a digital dynamometer (TKK 5401 GRIP D; Takei, Japan) and isokinetic muscle strength was measured using an isokinetic dynamometer on the preferred leg (Humac Norm by CSMi, Computer Sports Medicine, Inc., Stoughton, USA).

### Statistical analysis

Only participants who completed the study were included in the analysis (per-protocol). Missing data were not imputed; only participants with complete pre- and post-intervention data were analyzed. We tested the normality of the data using the Shapiro–Wilk test. To compare change scores across groups, we conducted a one-way ANOVA, while repeated measures ANOVA was used to assess pre-to-post changes in Metrnl and inflammatory markers within and between groups over time. When a significant group-by-time interaction was detected, Bonferroni post hoc adjustments were applied for multiple comparisons. We used Spearman’s rank correlation to examine associations between changes in Metrnl, inflammatory markers, and functional performance. To determine whether changes in serum Metrnl levels were predicted by these factors, we performed simple linear regression analysis. Statistical analyses were conducted using IBM SPSS Statistics macOS 29.0.2 software (IBM, Armonk, NY, USA), and all graphs were prepared using GraphPad Prism version 9.5.0 (GraphPad Software, San Diego, CA, USA). Statistical significance was set at *p* < 0.05.

## Results

Of the 90 participants recruited, 11 withdrew due to difficulty adhering to the intervention (n = 3), illness (n = 4), or personal reasons (n = 4). The final analysis included 79 participants (mean age: 73.8 ± 5.0 years; CG: n = 27, WG: n = 27, RWG: n = 25) who completed the intervention. Participant demographics are summarized in Table 1. Adherence to resistance training in the RWG was 91.3%. Participants in the RWG and WG exceeded their minimum age-adjusted weekly step goals, achieving 105% and 106%, respectively. There was no significant difference in caloric expenditure between the two exercise groups throughout the intervention, indicating comparable exercise volumes in both RWG and WG [35].

**Table 1:** Demographic characteristics of participants

Variables	CG (n = 27)	WG (n = 27)	RWG (n = 25)	*p*
Age, years	74.30 ± 5.87	73.85 ± 4.20	73.04 ± 4.63	0.66
*Gender*				0.16
Male, n (%)	8 (29.6)	12 (44.4)	5 (20)	
Female, n (%)	19 (70.4)	15 (55.6)	20 (80)	
Height, cm	156.17 ± 8.94	159.27 ± 9.80	157.70 ± 6.22	0.43
BMI, kg/m^2^	24.66 ± 4.52	24.10 ± 2.24	23.44 ± 2.79	0.43
Serum Metrnl, pg/mL	272.87 ± 78.39	268.28 ± 40.15	245.89 ± 39.51	0.19
Skeletal muscle mass, kg	21.11 ± 4.94	22.27 ± 4.64	20.68 ± 3.10	0.40
PA volume, MET-min/wk	1071.11 ± 925.02	1688.23 ± 1056.34	1235.20 ± 959.54	0.06
Stroop Interference, score	− 0.67 ± 8.70	− 1.26 ± 9.92	− 2.63 ± 8.81	0.70
Gait speed, m/s	1.04 ± 0.15	1.11 ± 0.22	1.07 ± 0.18	0.42
VO_2_ peak, ml/kg/min	20.55 ± 4.39	20.72 ± 3.77	20.32 ± 3.63	0.93
Hyperlipidemia, n (%)	2 (7.4)	4 (14.8)	4 (16.0)	0.60
Hypertension, n (%)	9 (33.3)	8 (29.6)	7 (28.0)	0.91
Diabetes Mellitus, n (%)	2 (7.4)	5 (18.5)	3 (12.0)	0.47

CG, active control group; WG, walking group; RWG, combined resistance and walking group; M, male; F, female; BMI, body mass index; Metrnl, meteorin-like protein; PA, physical activity

Values are presented as mean ± standard deviation, with statistical significance set at *p* < 0.05

### Exercise increased serum Metrnl levels

At baseline, serum Metrnl levels ranged from 165.10 to 438.62 pg/mL. Following the 12-week intervention, both the RWG and the WG showed significant increases in Metrnl levels, while the CG exhibited a decline. Specifically, the RWG increased from 245.89 ± 39.51 to 269.01 ± 50.55 pg/mL (9.4%, *p* = 0.016, Cohen’s *d* = 0.52), the WG from 268.28 ± 40.15 to 294.54 ± 58.49 pg/mL (9.8%, *p* = 0.009, Cohen’s *d* = 0.63), and the CG decreased from 272.87 ± 78.39 to 248.70 ± 53.11 pg/mL (− 8.9%, *p* = 0.060, Cohen’s *d* = 0.38).

**Fig. 2:**
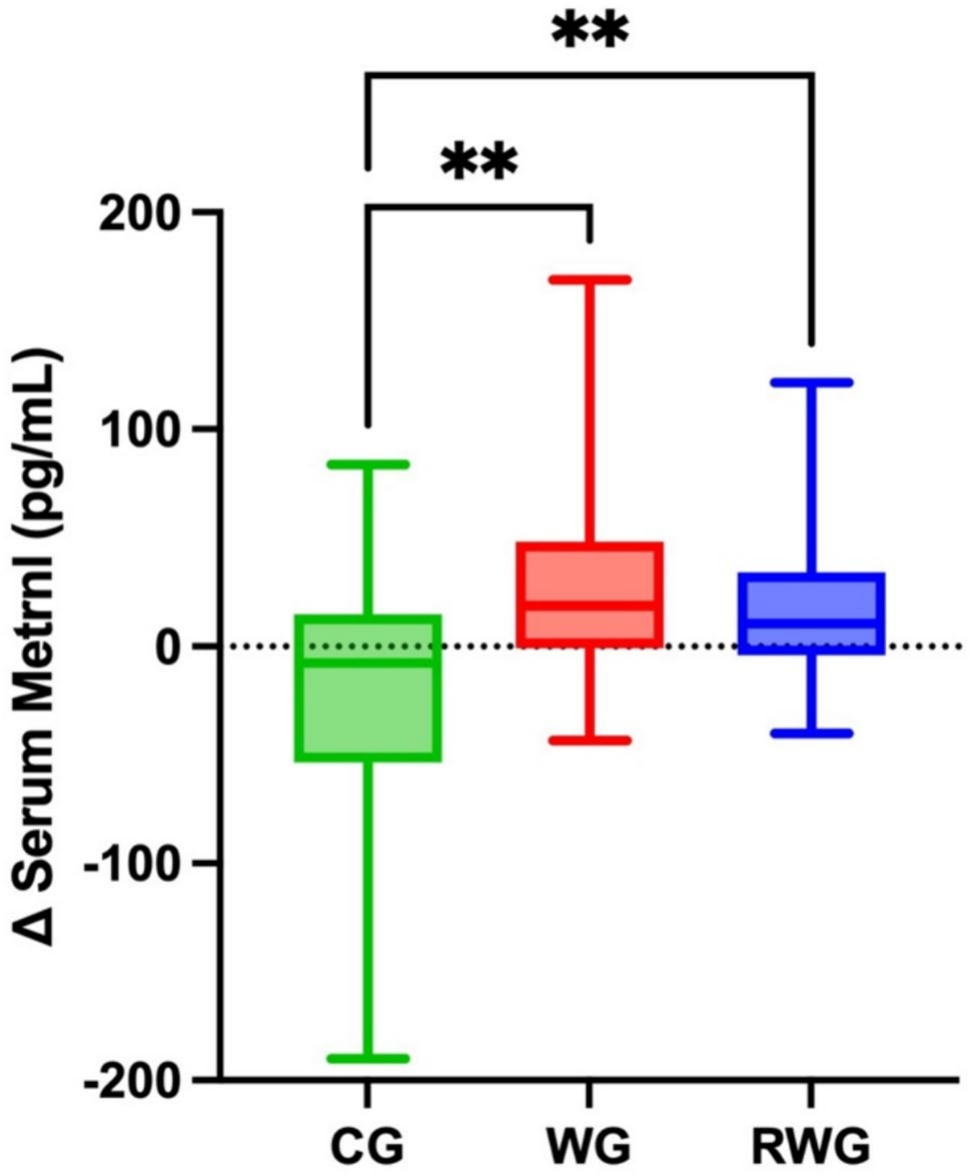
Changes (Δ) in serum Metrnl levels after the 12-week intervention. Metrnl levels increased following the 12-week exercise intervention compared to the control. CG, active control group; WG, walking group; RWG, combined resistance and walking group. Error bars represent standard deviations. Between-group statistical significance is denoted as **p ≤ 0.01

A significant group-by-time interaction was found (*F*(2,76) = 8.12, *p* < 0.001), indicating that changes in Metrnl levels differed significantly among groups (Fig. 2). Post hoc comparisons of the change scores revealed that both exercise groups showed significantly greater increases compared to the control group (WG vs. CG: *M*_diff_ = 50.44, *p* = 0.002; RWG vs. CG: *M*_diff_ = 47.29, *p* = 0.004).

### Exercise reduced inflammatory cytokines

We observed significant group-by-time interactions for all inflammatory cytokines (Table 2). In the RWG, IL-6 levels significantly declined after the 12-week intervention (*p* < 0.001, Cohen’s *d* = 1.00), with post hoc analysis revealing a significant difference compared to the WG (M_diff_ = 0.61, *p* = 0.022). TNF-α levels decreased significantly in both the WG (*p* < 0.001, Cohen’s *d* = 0.91) and RWG (*p* = 0.002, Cohen’s *d* = 0.71) but increased in the CG (*p* = 0.002, Cohen’s *d* = 0.65). CRP levels significantly declined in the WG (*p* = 0.012, Cohen’s *d* = 0.91) but increased in the CG (*p* = 0.044, Cohen’s *d* = 0.65). Additionally, post hoc analysis indicated a significant difference between the RWG and CG (M_diff_ = − 1.79, *p* = 0.041) after the intervention.

**Table 2:** Effects of 12-week intervention on level of inflammatory cytokines IL-6, TNF-α and CRP

Variable	Group	Baseline	Post	*M*_diff_	Group * Time Interaction			Post-hoc Pairwise Comparisons
		*M* ± SD	*M* ± SD		*F*	*p*	*η*^2^	
IL-6 (pg/mL)	CG	2.52 ± 2.50	2.63 ± 3.59	0.11	9.440	< 0.001	0.20	WG versus RWG: *M*_diff_ = 0.61,* p* = 0.022
	WG	2.88 ± 1.28	2.85 ± 2.21	− 0.03				
	RWG	2.60 ± 3.42	2.24 ± 4.29	− 0.36*				
TNF-α (pg/mL)	CG	8.23 ± 3.94	9.17 ± 4.96	0.94*	19.968	< 0.001	0.34	-
	WG	7.46 ± 3.29	6.76 ± 2.96	− 0.70*				
	RWG	8.22 ± 4.37	7.66 ± 4.26	− 0.57*				
CRP (mg/L)	CG	2.44 ± 2.90	3.21 ± 3.64	0.77	6.433	0.003	0.15	RWG versus CG: *M*_diff_ = − 1.79, *p* = 0.041
	WG	2.60 ± 1.91	1.87 ± 2.00	− 0.74*				
	RWG	2.41 ± 2.71	1.42 ± 1.43	− 0.98*				

Values are presented as mean (M) ± standard deviation (SD)

CG, active control group; WG, walking group; RWG, combined resistance and walking group; M_diff_, mean difference; IL-6, interleukin-6, TNF-α, tumor necrosis factor-alpha, CRP: C-reactive protein. Post-hoc results are based on Bonferroni's correction. Statistically significant differences (*p* ≤ 0.05) are indicated by an asterisk

### Metrnl linked to functional capacity in the combined resistance and walking group

**Fig. 3:**
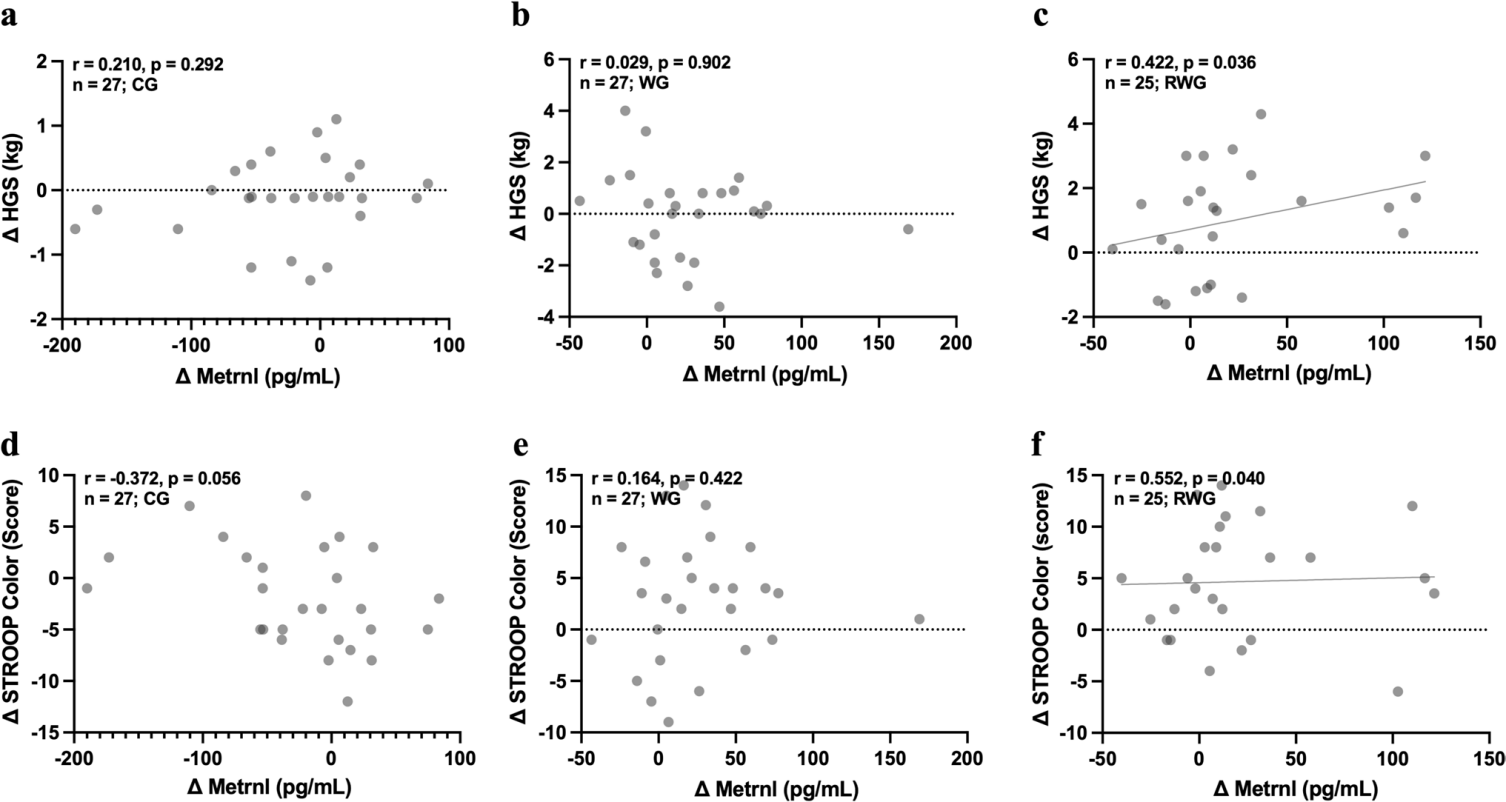
Correlation of changes (Δ) in Metrnl with functional capacity variables across all intervention groups. Positive correlations between Metrnl and **a–c** HGS, as well as **d–f** Stroop color score were observed only in the RWG. CG, active control group; WG, walking group; RWG, combined resistance and walking group; Metrnl, Meteorin-like protein; HGS, handgrip strength

When examining the association between serum Metrnl levels, inflammation, and functional capacity within each intervention group, we did not find significant correlations between Metrnl and inflammatory biomarkers. However, in the RWG, we observed significant positive correlation between changes in Metrnl and HGS (*r* = 0.422, *p* = 0.036; Fig. 3c) and Stroop color test performance (*r* = 0.552, *p* = 0.040; Fig. 3f). No significant correlations were identified in the WG. When analyzing changes in body composition measured by Bioelectrical Impedance Analysis (BIA), we found a significant positive correlation with muscle mass within CG (*r* = 0.459, *p* = 0.028), and a negative correlation with fat mass within RWG (*r* = − 0.501, *p* = 0.021). Detailed results of BIA outcomes are provided in the supplementary materials.

### Metrnl linked to inflammation and functional capacity independent of exercise group allocation

**Fig. 4:**
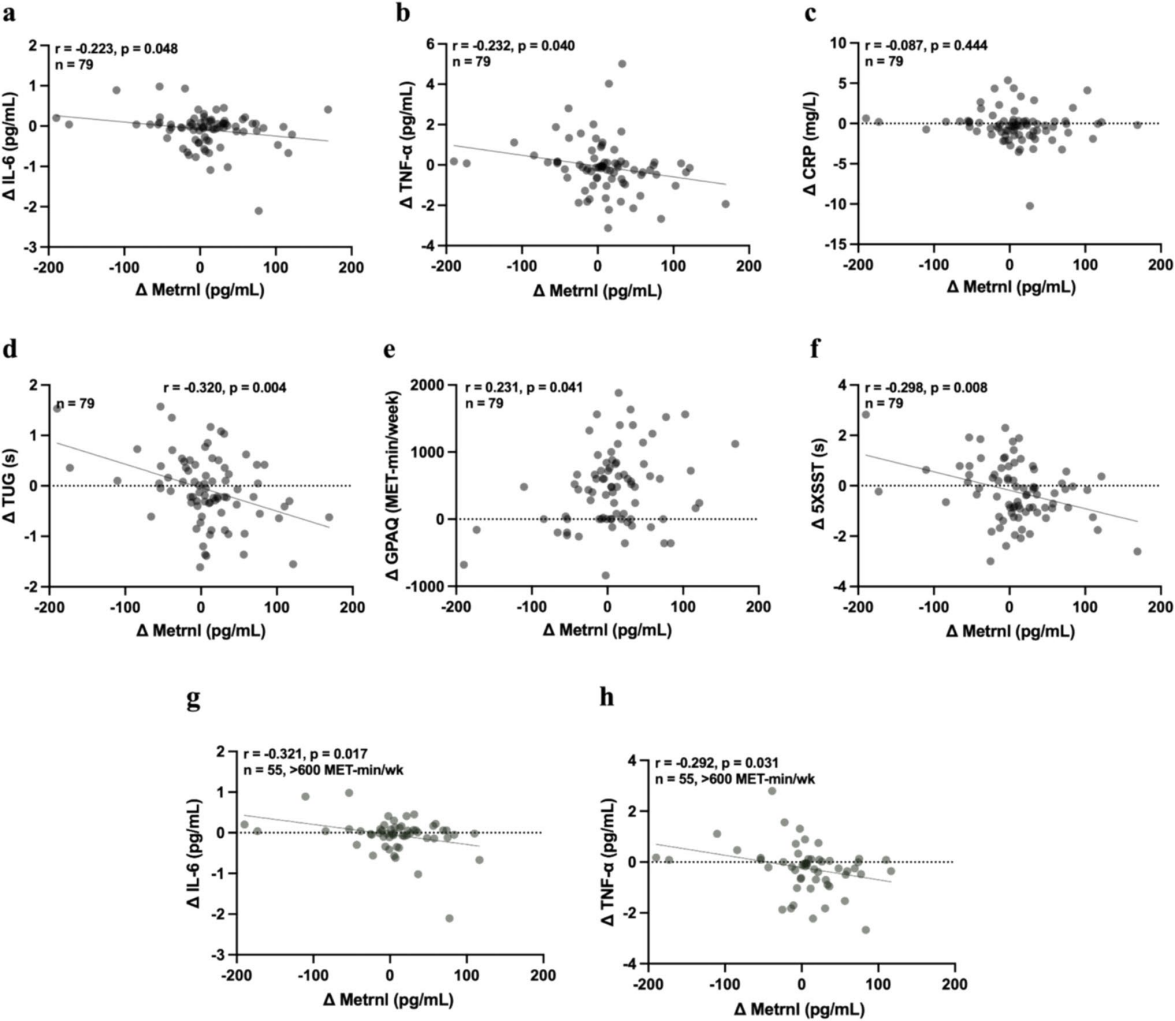
Correlation of changes (Δ) in Metrnl with inflammatory and functional capacity variables across all participants, irrespective of exercise group allocation. **a–f** Present the association of Metrnl with **a** interleukin-6 (IL-6), **b** tumor necrosis factor-alpha (TNF-α), **c** C-reactive protein (CRP), **d** timed up and go (TUG), **e** physical activity level based on Global Physical Activity Questionnaire (GPAQ), and **f** 5 times sit-to-stand test (5** × **SST). **g, h** Associations observed specifically among participants with moderate physical activity levels (≥ 600 MET-min/week). Changes in Metrnl were negatively associated with **g** IL-6 and **h** TNF-α

To further explore the relationship between serum Metrnl levels and relevant factors, we analyzed associations with inflammatory markers and functional performance across all participants, regardless of group allocation. Changes in serum Metrnl levels were significantly associated with IL-6 (*r* = − 0.223, *p* = 0.048; Fig. 4a) and TNF-α (*r* = − 0.232, *p* = 0.040; Fig. 4b) levels (both showing a negative trend) and the GPAQ-based volume of PA (*r* = 0.231, *p* = 0.041; Fig. 4e). Additionally, improvements in TUG (*r* = − 0.320, *p* = 0.004; Fig. 4d) and 5** × **SST (*r* = − 0.298, *p* = 0.008; Fig. 4f) performances were positively associated with increased Metrnl levels in older adults (Fig. 4).

In addition, we categorized participants based on their baseline volume of PA, using the World Health Organization’s threshold of 600 MET-min/week to distinguish between moderately active and sedentary individuals. Those classified as moderately active at baseline exhibited stronger negative correlations between changes in Metrnl and both IL-6 (*r* = − 0.321, *p* = 0.017; Fig. 4g) and TNF-α (*r* = − 0.292, *p* = 0.031; Fig. 4h).

### Predictors of Metrnl levels

To examine the individual contributions of each variable to changes in serum Metrnl levels, we conducted simple linear regression analyses, with each predictor entered into the model separately. As shown in Table 3, the results indicated that WG (β = 0.237, *p* = 0.035), TNF-α (β = − 0.232, *p* = 0.040), IL-6 (β = − 0.223, *p* = 0.048), MET-min/week (β = 0.305, *p* = 0.006), 5** × **SST (β = − 0.354, *p* = 0.001), and the TUG (β = − 0.379, *p* < 0.001) were significant predictors of changes in Metrnl levels. Among them, TUG emerged as the strongest predictor, explaining 14.4% of the variance in Metrnl levels, followed by 5XSTT (12.5%) and MET-min/week (9.3%). WG group showed a moderate effect (β = 0.24, *p* = 0.035), in contrast, the WRG was not a significant predictor (β = 0.185, *p* = 0.102). Furthermore, no other variables related to physical or cognitive function outcomes were found to significantly predict Metrnl levels.

**Table 3:** Simple linear regression for predictors of changes in serum Metrnl levels

Predictor	B	β	*p*	R^2^ (%)
WG	27.70	0.24	0.035	5.6
Δ MET-min/week	0.03	0.31	0.040	9.3
Δ TNF-α	− 10.04	− 0.23	0.048	5.4
Δ IL-6	− 28.49	− 0.22	0.006	5.0
Δ 5** × **SST	− 17.05	− 0.35	0.001	12.5
Δ TUG	− 30.85	− 0.379	< 0.001	14.4

B, Unstandardized coefficient; β, standardized coefficient; WG, walking group; MET, metabolic equivalent; TNF-α, tumor necrosis factor-alpha; IL-6, interleukin-6; 5** × **SST, 5 times sit to stand test, TUG, timed up and go. Statistically significant differences (*p* ≤ 0.05)

## Discussion

Despite the growing interest in Metrnl as a potential biomarker and therapeutic target, human studies, particularly in older populations, are limited. To the best of our knowledge, this is the first study to investigate the effects of exercise on circulating Metrnl levels in older adults. We initially hypothesized that combined exercise would produce greater increases in serum Metrnl levels and stronger associations with improvements in inflammatory markers and functional outcomes compared to walking alone. However, our results indicated that while both exercise interventions differed significantly from the active control group, no significant difference was observed between the two exercise modalities. Correlation analyses revealed that reductions in pro-inflammatory markers such as IL-6 and TNF-α were associated with increases in Metrnl levels. Additionally, changes in Metrnl were significantly related to volume of PA (assessed via GPAQ), physical and cognitive performance (TUG, 5** × **SST, HGS, and Stroop color test), as well as body composition (fat and muscle mass). Furthermore, linear regression analyses identified TUG, 5** × **SST, PA volume, TNF-α, and IL-6 as significant predictors of changes in Metrnl levels. Among the exercise interventions, walking exercise significantly contributed to changes in Metrnl levels. Although these correlations were statistically significant, the strength of the associations was modest, suggesting that Metrnl response to exercise may be influenced by multiple interacting factors rather than a single determinant. These findings suggest that both inflammatory and functional factors may influence Metrnl response to exercise in older adults.

Preclinical studies have demonstrated that Metrnl may mediate the beneficial effects of exercise on metabolic health by modulating inflammatory pathways and enhancing energy expenditure [32, 43–47]. However, human studies remain limited, with most research focusing on the acute effects of exercise in young populations [30–32]. In our study, both walking and combined exercise groups showed significantly higher Metrnl levels compared to controls after 12 weeks (Fig. 2), aligning with findings by Bonfante et al., who reported similar improvements following a 16-week combined program in overweight adults with type 2 diabetes [34]. While Liu and colleagues demonstrated that exercise intensity influences Metrnl secretion [48], emerging evidence also suggests that different modes of muscle contraction may differentially regulate Metrnl [25]. The mechanisms underlying Metrnl responses to different exercise modalities have not yet been thoroughly investigated. Aerobic exercise appears to stimulate Metrnl via oxidative metabolism and AMPK-PGC-1α1 signaling [49], whereas resistance training may do so through AMPK-independent pathways related to muscle damage and hypertrophy [25, 32]. This is further supported by Baht and colleagues, who identified Metrnl as a key player in muscle regeneration following injury [20].

Our findings contribute to the growing body of evidence supporting a negative association between Metrnl and inflammatory cytokines, specifically IL-6 and TNF-α (Fig. 3a, b), but not CRP. This pattern underscores the potential role of Metrnl in the aging through its anti-inflammatory effects and involvement in muscle regeneration. While El-Ashmawy and colleagues reported significant associations with all three markers, including high-sensitivity CRP (hsCRP) [8], the discrepancy may arise from differences in CRP assay sensitivity, highlighting the importance of marker selection in interpreting inflammatory responses. Furthermore, we found that fat mass was negatively associated with Metrnl levels in the WRG participants, a relationship potentially linked to inflammation (Fig. 4b). Although our results did not mirror the impact of exercise on this relationship, the negative correlation was significantly more pronounced in participants with moderate PA levels (Fig. 4), consistent with previous studies showing enhanced health benefits in physically active older adults [50]. These findings suggest that higher baseline activity may potentiate Metrnl’s anti-inflammatory response. These findings in older adults align with preclinical evidence demonstrating Metrnl’s critical role in muscle regeneration and immune regulation, reinforcing its relevance in mitigating age-related functional decline. Metrnl facilitates muscle repair by stimulating the expansion of muscle stem cells and regulating inflammation. It does so by coordinating macrophage-derived TNF-induced fibro/adipogenic progenitor (FAP) apoptosis through the STAT3–IGF-1 signaling axis, thereby promoting a return to homeostasis [24, 26].

So far, no clinical studies have investigated the association between exercise-induced Metrnl and functional capacity. Although Recent research by Wang and colleagues strongly supports the clinical relevance of Metrnl, showing that lower serum levels (< 197.2 pg/mL) are associated with a greater risk of sarcopenia, and are correlated with key sarcopenia-related measures such as appendicular skeletal muscle mass, HGS, and gait speed [27]. These findings underscore Metrnl’s regenerative potential, though further research is needed to confirm its role as a biomarker for age-related conditions. Although our participants were not sarcopenic, we also observed associations between changes in Metrnl and physical function with HGS, while 5** × **SST and TUG emerged as strong predictors of Metrnl changes. These results suggest that tracking these parameters alongside Metrnl may help prevent the onset of sarcopenia. Furthermore, we identified volume of PA, IL-6, and TNF-α as moderate predictors of Metrnl changes (Table 3). Despite relatively low R^2^ values, we used this analysis to screen variables with meaningful associations for potential inclusion in future multivariable models.

As is common in human intervention studies, it was not possible to achieve complete control over all variables; however, every effort was made to minimize potential confounding factors. Although the randomized controlled design strengthens internal validity, the modest sample size (n = 79) may limit statistical power for detecting small or subgroup-specific effects. The study population, comprising community-dwelling older adults from a single region, may reduce generalizability to broader or more clinically diverse populations. No significant differences in Metrnl levels were observed between the walking and combined exercise groups, possibly due to the moderate intensity or the influence of total physical activity volume rather than modality. Future studies should explore higher intensities, varied training loads, or longer durations to detect potential modality-specific effects. The control group, which received health education and had relatively high baseline activity levels, was not strictly monitored, potentially diluting group differences. Rigorous physical activity monitoring or inclusion of a sedentary control.

## Conclusion

Our study provides the first evidence that a 12-week exercise program, involving either walking alone or walking in combination with resistance training, significantly increased Metrnl levels in older adults compared with active controls. These findings demonstrate that Metrnl is linked to key markers of inflammation (IL-6 and TNF-α), moderate level of PA, physical function (TUG, 5** × **SST, and HGS), cognitive performance (Stroop color test), and body composition (fat and muscle mass). These results suggest that Metrnl may serve as a biomarker connecting exercise, inflammation, and functional capacity in older adults. Despite limitations such as a modest sample size, lack of detailed comparisons between exercise modalities, and uncontrolled variables like medication use, the findings offer important insights into the role of Metrnl in aging. The observed exercise-induced increase in Metrnl, which is associated with reduced inflammation and improved physical and cognitive function, suggests its potential as a therapeutic target to enhance training responses and support healthy aging.

## Supplementary Information

Below is the link to the electronic supplementary material.

Supplementary file1 (PDF 121 KB)

## Data Availability

Due to ethical and legal restrictions, the data underlying this study cannot be made publicly available. De-identified data may be shared upon reasonable request and approval by the IRB.
