# Supplementary material for: Effects of 12-week exercise on Meteorin-like levels, inflammation, and functional capacity in older adults: Korean national aging project randomized controlled study
Source: Eur Geriatr Med. 2025 Jul 23;16(6):2129–40. doi: 10.1007/s41999-025-01272-2 (PMC12743662; doi:10.1007/s41999-025-01272-2)
Supplement: Supplementary file 1 — Supplementary file1 (PDF 121 KB) [file 41999_2025_1272_MOESM1_ESM.pdf]

[Supplementary Figure]

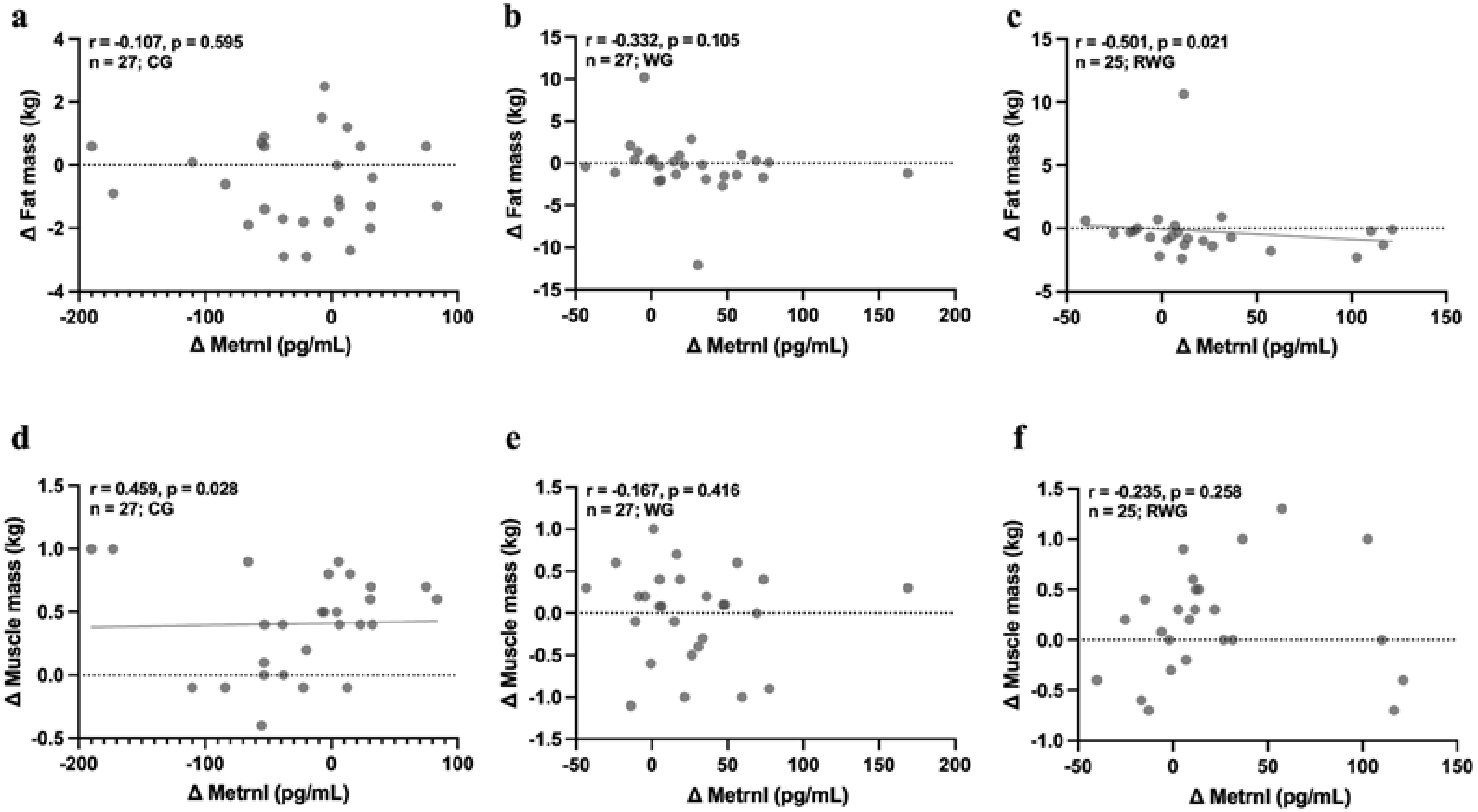

Supplementary. Correlation of changes (Δ) in Metrnl with body composition measured by Bioelectrical Impedance Analysis (BIA) across all intervention groups. *Changes in Metrnl positively linked to fat mass (c) in the RWG and muscle mass (d) in the CG. Abbreviations: CG, active control group; WG, walking group; RWG, combined resistance and walking group; Metrnl, Meteorin-like protein.*
